# Evaluation of an intelligent artificial climate chamber for high-throughput crop phenotyping in wheat

**DOI:** 10.1186/s13007-022-00916-9

**Published:** 2022-06-07

**Authors:** Anhua Ren, Dong Jiang, Min Kang, Jie Wu, Fangcheng Xiao, Pei Hou, Xiuqing Fu

**Affiliations:** 1 College of Engineering, Nanjing Agricultural University, Nanjing, 210031 China; 2 Plant Phenomics Research Center, Nanjing Agricultural University, Nanjing, 210095 China; 3 Jiangsu Key Laboratory of Intelligence Agricultural Equipment, Nanjing, 210031 China; 4 Nanjing Huitong Crop Phenotypic Research Institute Co., Ltd, Nanjing, 211225 China

**Keywords:** Intelligent artificial climate chamber, Environmental Control System, Phenotype Acquisition System, Crop phenotype acquisition system, Wheat cultivation test

## Abstract

**Background:**

The superposition of COVID-19 and climate change has brought great challenges to global food security. As a major economic crop in the world, studying its phenotype to cultivate high-quality wheat varieties is an important way to increase grain yield. However, most of the existing phenotyping platforms have the disadvantages of high construction and maintenance costs, immobile and limited in use by climatic factors, while the traditional climate chambers lack phenotypic data acquisition, which makes crop phenotyping research and development difficult. Crop breeding progress is slow. At present, there is an urgent need to develop a low-cost, easy-to-promote, climate- and site-independent facility that combines the functions of crop cultivation and phenotype acquisition. We propose a movable cabin-type intelligent artificial climate chamber, and build an environmental control system, a crop phenotype monitoring system, and a crop phenotype acquisition system.

**Result:**

We selected two wheat varieties with different early vigor to carry out the cultivation experiments and phenotype acquisition of wheat under different nitrogen fertilizer application rates in an intelligent artificial climate chamber. With the help of the crop phenotype acquisition system, images of wheat at the trefoil stage, pre-tillering stage, late tillering stage and jointing stage were collected, and then the phenotypic information including wheat leaf area, plant height, and canopy temperature were extracted by the crop type acquisition system. We compared systematic and manual measurements of crop phenotypes for wheat phenotypes. The results of the analysis showed that the systematic measurements of leaf area, plant height and canopy temperature of wheat in four growth periods were highly correlated with the artificial measurements. The correlation coefficient (r) is positive, and the determination coefficient (*R*^*2*^*)* is greater than 0.7156. The root mean square error (*RSME*) is less than 2.42. Among them, the crop phenotype-based collection system has the smallest measurement error for the phenotypic characteristics of wheat trefoil stage. The canopy temperature *RSME* is only 0.261. The systematic measurement values of wheat phenotypic characteristics were significantly positively correlated with the artificial measurement values, the fitting degree was good, and the errors were all within the acceptable range. The experiment showed that the phenotypic data obtained with the intelligent artificial climate chamber has high accuracy. We verified the feasibility of wheat cultivation and phenotype acquisition based on intelligent artificial climate chamber.

**Conclusion:**

It is feasible to study wheat cultivation and canopy phenotype with the help of intelligent artificial climate chamber. Based on a variety of environmental monitoring sensors and environmental regulation equipment, the growth environment factors of crops can be adjusted. Based on high-precision mechanical transmission and multi-dimensional imaging sensors, crop images can be collected to extract crop phenotype information. Its use is not limited by environmental and climatic factors. Therefore, the intelligent artificial climate chamber is expected to be a powerful tool for breeders to develop excellent germplasm varieties.

**Supplementary Information:**

The online version contains supplementary material available at 10.1186/s13007-022-00916-9.

## Introduction

The combination of climate change and the new crown epidemic has brought huge challenges to food security in China and the world [1, 2]. The foundation for coping with challenges and ensuring national food security involves the analysis of the regulation mechanism of crop gene and phenotype formation, selection of new varieties with high yield, high quality, are green and stress resistance; realisation of precision cultivation and fine breeding methods and improvement of the utilisation efficiency of crop germplasm resources [3, 4]. High-throughput crop-phenotype acquisition is the key to in-depth interpretation of gene functions and breaking through the bottleneck of precision breeding technology. Studies should focus on the laws of crop growth and development, reveal gene regulation pathways and optimise precision management of crop cultivation and acceleration of crop improvement [5]. The phenotype acquisition platform is an important hardware basis for rapid screening of germplasm resources, phenotype identification, and formation mechanism research [6–8], and it is mainly composed of mechanical devices or drones equipped with sensors [9, 10].

The traditional artificial climate chamber is a key place for the cultivation of crop varieties, and its functions mostly focus on the control of environmental parameters. Guo Minghang et al. developed a scientific research-type artificial arid climate chamber, which can simulate main environmental factors such as light, temperature, and CO_2_ concentration [11]. Guo Zhuangliang et al. designed an environmental data acquisition system based on the CAN bus for an artificial climate chamber, which collects and transmits information on plant growth environmental factors through various sensors to achieve the purpose of environmental control [12]. Zhang Xinyu et al. designed an artificial climate chamber based on a Field-Programmable Gate Arrays (FPGA) environmental control system, and realized the automatic adjustment of the Photosynthetic Photon Flux Density (PPFD) and Red Photon Flux Density (RPFD)/Blue Photon Flux Density (BPFD) (R/B) of the plant canopy with the help of multi-channel photonic sensors [13]. Since artificial climate chambers generally do not have the function of high-throughput acquisition of crop phenotype data, most of the phenotype acquisition work still needs to be done manually, which has shortcomings such as low efficiency and large errors.

As phenomics has gradually become a recognized research hotspot in the frontier of life sciences, the crop phenotyping platform integrating precise management of crop cultivation, high-precision mechanical transmission, and multi-sensor data collection and analysis has become the development trend of high-throughput phenotype acquisition [14].In recent years, crop phenotyping research platforms have been greatly developed, such as the Plant Monitor developed by the French Academy of Agricultural Sciences, which uses artificial growth boxes equipped with RGB, infrared and fluorescence imaging units, but is limited by the small number of monitoring, large workload and low efficiency [15]. The Pheno Watch Crop 3D developed by the Institute of Botany of the Chinese Academy of Sciences integrates various imaging units such as lidar and high-resolution cameras, and realizes the extraction of three-dimensional information for the first time, with a high degree of automation. However, the phenotypic data obtained differs from the actual crop condition due to the differences between the greenhouse environment and the outdoor environment [16]. At present, there are many types of platforms for phenotypic measurement, but most of them are geographically restricted, and equipment maintenance costs are high in the later period [17]. There is a lack of multi-functional phenotype monitoring equipment for comprehensive environmental condition control, image acquisition and phenotype acquisition, and it is difficult to meet the actual needs of current crop phenotype research to obtain relevant phenotype data on the impact of biotic or abiotic factors on crop yield [18].

In order to break through the bottleneck of phenotyping research and provide more powerful phenotypic data support for promoting the wheat breeding process, a relatively low-cost, high-throughput, easy-to-promote and easy-to-promote high-throughput method that is not limited by the environment and climate is urgently needed. Phenotype acquisition platform. Therefore, based on the traditional artificial climate chamber and combined with the environmental control, phenotype monitoring and phenotype acquisition systems, this article carried out the research and development of an intelligent artificial climate chamber with crop cultivation management and phenotype acquisition functions during the whole growth period of wheat. The precision cultivation test of wheat realised the continuous acquisition of phenotypic characteristics during wheat growth, improved the lack of phenotypic feature extraction and analysis in the traditional artificial climate chamber and verified the feasibility of the artificial climate chamber for crop phenotyping research. The purpose of our research is to develop an intelligent artificial climate chamber that can simulate the environmental conditions of crop growth and obtain phenotypic information, so as to help breeders develop high-quality, high-yield, high-tolerance crop varieties and ensure world food production security.

## Materials and methods

### Experimental setup

**Fig. 1:**
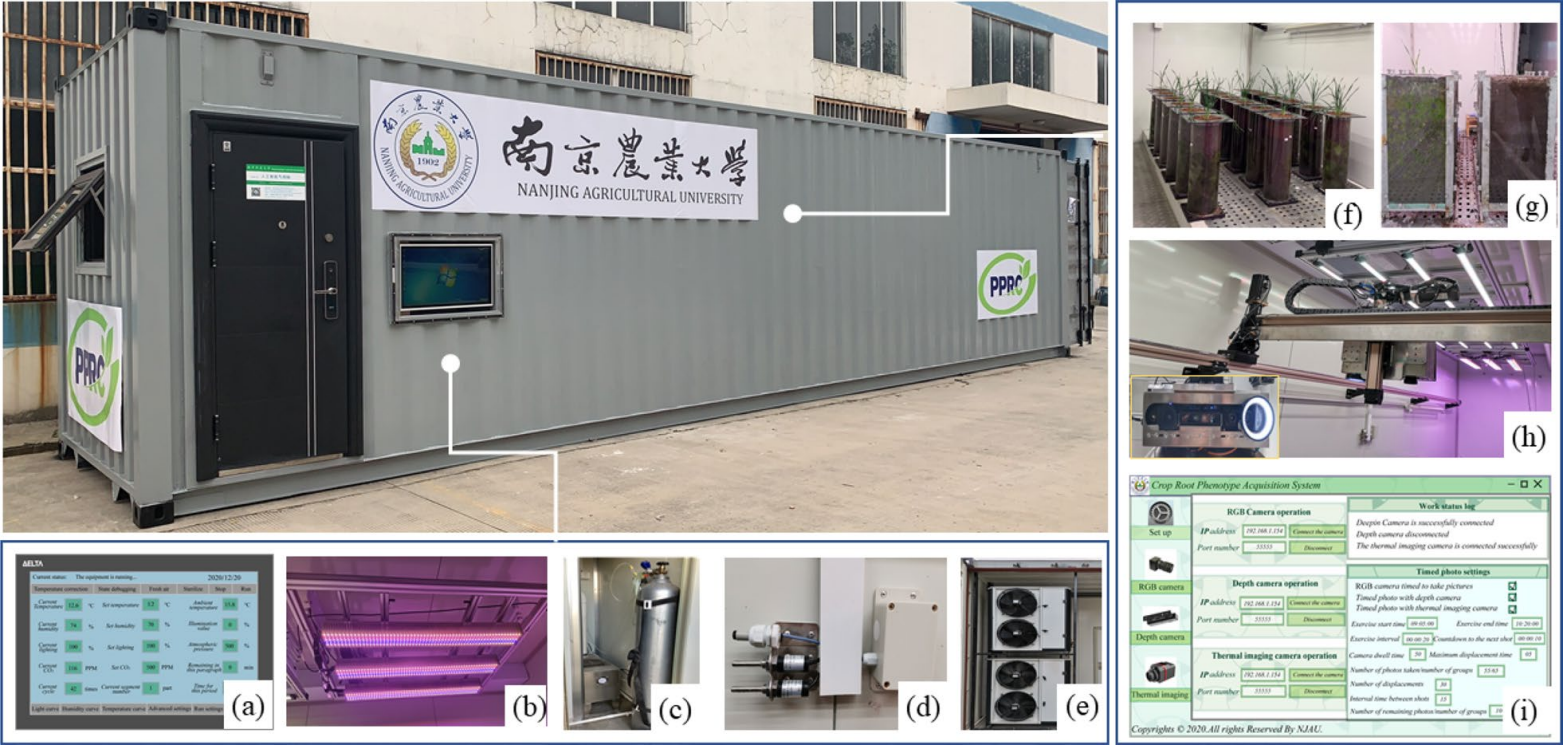
Schematic of the overall structure of the intelligent artificial climate chamber

The experiment was carried out in the intelligent artificial climate chamber of the Baima Base of Nanjing Agricultural University. The test subjects were selected from the Qingnong 2(QN2) and Liangxing 77(LX77) wheat varieties of the State Key Laboratory of Crop Genetics and Germplasm Innovation. Salt-free coconut bricks were selected as the cultivation substrate for soilless cultivation. The wheat seeds were sown on the root support site of the round root box at a depth of 3 cm, and three repeat groups were set up (Fig. 1f). A total of 2 × 3 × 3 round root boxes were planted. Three grains of wheat are sown in each round root box. The nitrogen, phosphorus and potassium fertilisers used in the experiment were urea (containing N46%), superphosphate (containing P_2_O_5_18%) and potassium sulphate (containing K_2_O50%), respectively. The amount of nitrogen fertiliser was set as a variable, and the trefoil stage of wheat was fertilised and irrigated at the Pre-tillering stage, jointing stage and growing period. Additional file 1: Table S1 shows the environment and cultivation parameters in the crop cultivation and phenotype acquisition area.

### Development of intelligent artificial climate chamber

#### Composition of intelligent artificial climate chamber

Fig. 1 shows the developed intelligent artificial climate chamber. Fig. 1a–e display the environmental control system used to regulate the environmental parameters in the crop cultivation area of the chamber. Fig. 1f, g present the crop cultivation devices, namely, the flat root boxes and round root boxes, respectively, that were used for wheat cultivation experiments. Fig. 1h shows a crop phenotype monitoring system composed of a high-precision mechanical transmission device and the multiple sensors mounted on it. We obtained the phenotypic characteristics of the crop during the growth process. Fig. 1i shows the software interface of the crop phenotype acquisition system, which was used to analyse and process the image information of the crop and extract phenotypic characteristic parameters. The environmental parameters in the chamber were set by the control system. The crop phenotype monitoring system was used to obtain images and information of crops on the cultivation device, and the crop phenotypic characteristics were obtained based on the phenotype acquisition system.

#### Environmental control system

**Fig. 2:**
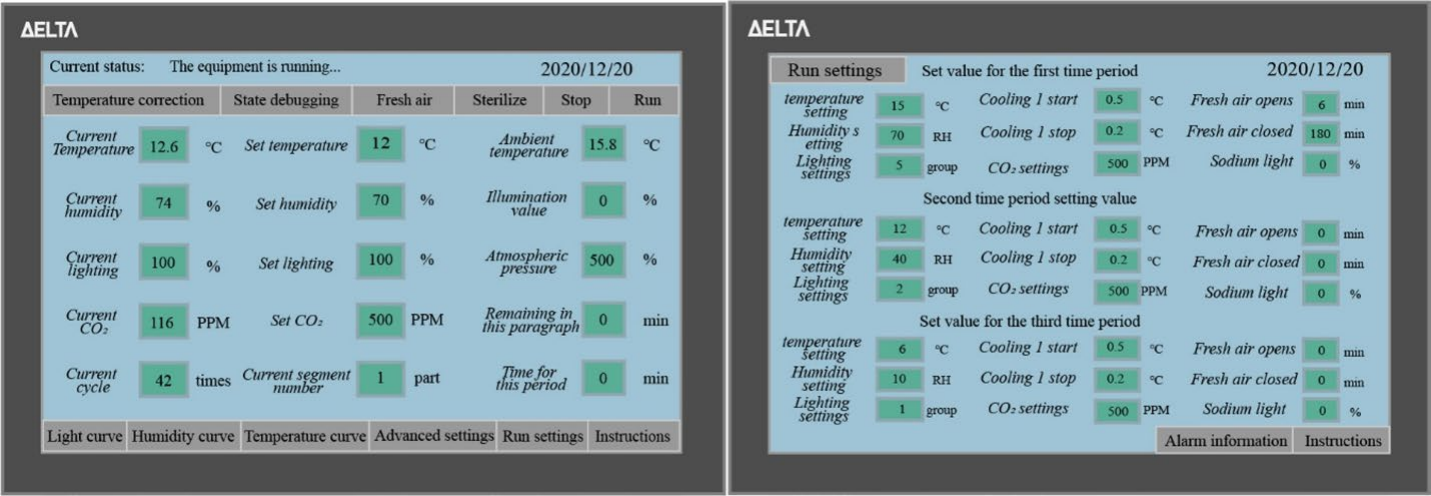
Environmental control system architecture

Fig. 2 shows the interface of the developed environmental control system. The control of environmental parameters, including the chamber temperature, humidity, light, CO_2_ concentration, air pressure can be realised through a touch screen, and the circulation ventilation inside and outside the chamber were attained. Additional file 1: Table S2 in exhibits the control range. The environmental control equipment of the intelligent artificial climate chamber mainly includes environmental factor perception equipment and environmental factor control equipment. Among them, the environmental sensing equipment mainly includes sensor equipment parameters such as temperature, humidity, carbon dioxide concentration and light intensity, as shown in Additional file 1: Table S3. Environmental control equipment includes air conditioners, humidifiers, dehumidifiers, carbon dioxide supply devices, plant growth lights and other equipment. The environmental factor sensor in the crop cultivation and phenotype acquisition area detects various environmental information in the chamber, and converts the real-time parameters into analog signals, and then the A/D converter converts the analog signals into digital signals and transmits them to the control core PLC, the PLC received signal is converted and displayed on the display screen, and the user can adjust the environmental factor parameters in the manual operation of the touch screen in the environmental control and analysis area. Then the PLC issues commands to the control equipment to control the environment in the chamber. Multiple control equipment operates independently without interference. Ventilation can set the start and end time to achieve the purpose of removing excess heat in the chamber and replacing fresh air.

#### Crop phenotype monitoring system

**Fig. 3:**
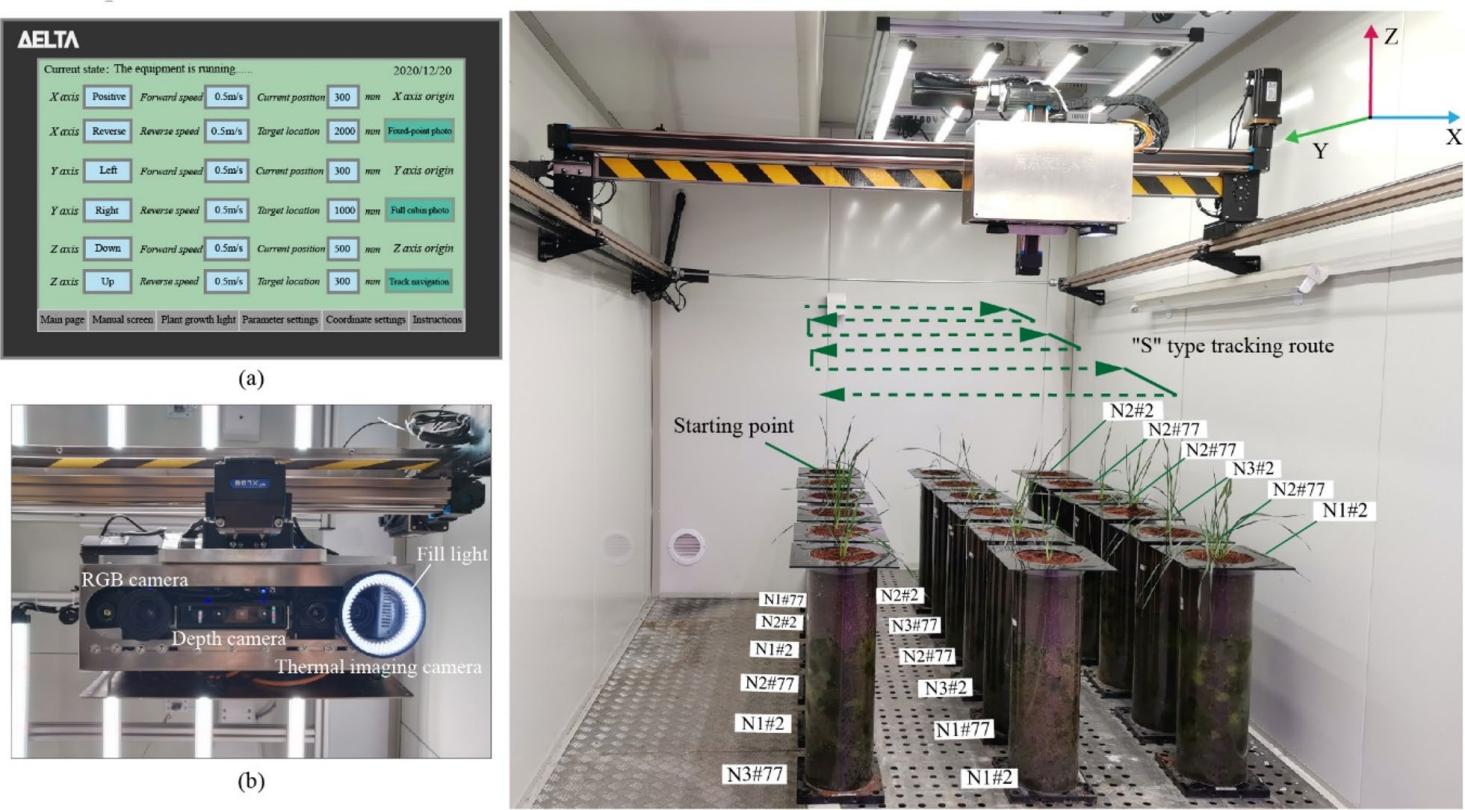
Hardware device of crop phenotype monitoring system

The crop phenotype monitoring system is mainly composed of high-precision mechanical transmission and multi-sensors. As shown in Fig. 3, the high-precision mechanical transmission mainly includes three parts: control, motion and auxiliary units. The upper computer in the control unit (Fig. 3a) can control the servo motor to move in the X/Y/Z directions in space, display the real-time position of the imaging device and communicate with the lower computer. The lower computer is responsible for executing the instructions issued by the upper computer, collecting the real-time position information of the servo motor, feeding back the position information to the upper computer, and controlling the position movement of the servo motor in space and the real-time adjustment of the speed in real time, so as to find the fault of the transmission device in time. Report to the host computer. The whole system adopts open-loop control. The key equipment parameters of the high-precision mechanical transmission are shown in Additional file 1: Table S4. Its operating range is: in the X-axis direction (0-6000 mm), in the Y-axis direction (0-2000 mm), and in the Z-axis direction (0-500 mm). The multi-element imaging sensor is used to collect crop image information, convert the optical signal into an electrical signal and transmit it to the core controller, which is the core device in the crop monitoring system. In order to meet the needs of obtaining the phenotypic parameters of crop leaf area, plant height, and canopy temperature, RGB cameras, depth cameras and thermal imaging cameras were selected to form a multi-element imaging sensor group (Fig. 3b). Based on the actual working conditions and the analysis of camera parameters, this paper selects industrial cameras with more reliable and stable performance, and their respective models and parameter information are summarized in: Additional file 1: Table S5. The imaging sensor sites are set according to the location of the cultivation area within the crop cultivation and phenotype acquisition zone, containing 6 × 3 (length x width) camera sites. The high-precision mechanical drive is equipped with a multi-element imaging sensor set. When the system is running, first of all, the control page of the high-precision mechanical drive is opened, and the position of the imaging device on the linear guide in the Z direction is adjusted so that the distance between the camera plane and the top of the root box is kept consistent at 0.6 m. Timed directional motion (moving speed of 0.5 m/s) is set. Control the mechanical drive to travel along the preset "S" shaped path, stay at each loci for 50 s, collect 3 to 5 pictures of wheat in each root box, and unify with the settings of camera timing photos in the subsequent crop phenotype monitoring system, which can realize the timing cruise monitoring of crops in the intelligent artificial climate chamber, and obtain RGB color images of wheat in a nondestructive way, depth images and infrared images to facilitate accurate extraction of canopy feature parameters.

#### Crop phenotype acquisition system

**Fig. 4:**
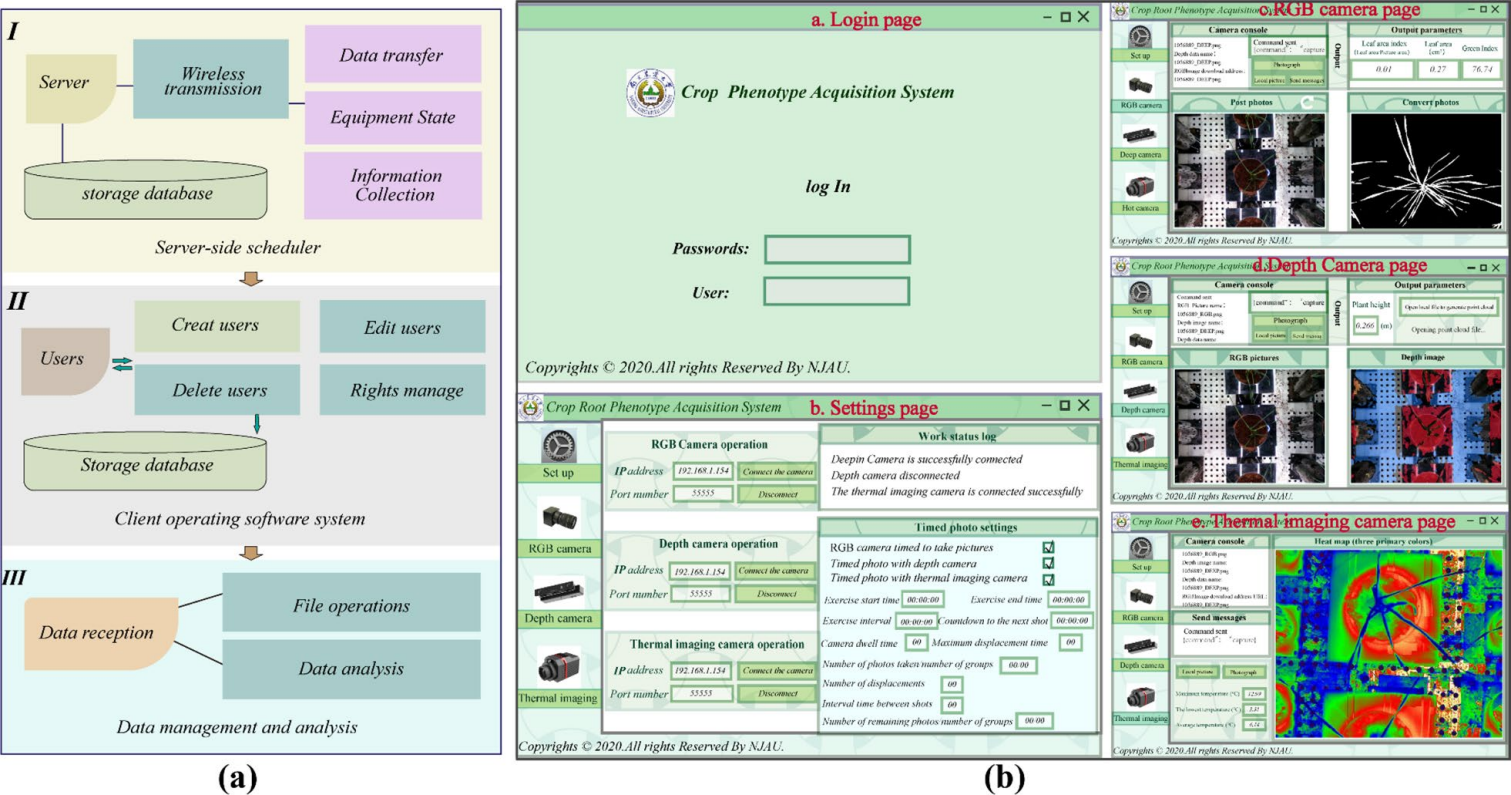
General diagram of the software system

The developed crop phenotype acquisition system was mainly used for crop image collection, phenotypic feature acquisition and data management. The software system adopts Client/Server control structure. C/S has the advantages of strong interactivity, secure data storage, low requirements for network traffic, and ready-to-use after installing the client. Based on the Visual Studio2017 development platform and programming languages ​​such as C#, we have completed the development of the server driver and the design of the client software interface. The server corresponds to the camera driver and is responsible for mobilizing the camera to capture images. According to the SDK software tool development kit provided by the camera manufacturer, based on the Linux ARM operating system and combined with the actual needs, we call the specific camera interface function API, set and control the camera-related parameters, write the camera driver, complete the secondary development, and realize the function of mobilizing the camera to capture images in real time. The client corresponds to the operation interface and provides a human–computer interaction interface. We develop user interface WPF based on Windows system under the integrated development environment of VS. We set the size and appearance of the software interface by adding a Form. We add classes and files, as well as controls such as buttons and labels on the dialog page, switch class items, set events or properties of the response controls in the solution manager, and call image recognition and processing function libraries at the same time to achieve different interfaces. Different function settings. The client and the server communicate with each other through Socket, and the two cooperate, coordinate and work together during operation, so as to realize the purpose of crop phenotype monitoring system to complete the functions of crop image acquisition, data analysis and characteristic parameter output. The image processing process is described in the next section. Fig. 4a shows the flow chart of the system function realisation. The server was used to store data and information collection; the user can perform image collection and data processing operations on the software client; the multiple data obtained by the imaging sensor can be wirelessly transmitted to the storage server, and they were used for post-processing and image processing. Fig. 4b shows the software interface of the system, where the login interface was used for user registration and login; the setting interface displayed the working status of the sensor and setting of the timing camera; the sensor interface displayed the operating status information, real-time collected crop images and extraction data. The phenotypic data of the crops can be obtained: leaf area size, leaf area index, green index, wheat individual plant height and minimum, maximum and average temperatures of the canopy of individual wheat.

#### Extraction of wheat phenotypic parameters based on multivariate imaging sensor 

##### Extraction of wheat phenotypic parameters based on RGB camera

The green index is the proportion of the green area in the wheat canopy image in the total image. The research standard in this paper is the ratio of the sum of green parts in the vertical projection direction of wheat canopy to the image. First, read the RGB image, check whether the image is uint8 data type, and count the total number of pixels of the RGB image. If the data type of the RGB image is uint8, the value range of each component image is [0 ~ 255]. Obtain the histogram of each color component of RGB image, and select the appropriate threshold for segmentation; Set the threshold of green component; Leave the green area image (G), remove the background, and output the green component value [19]. Dividing the green component value by the total number of image pixels is the green index for ratio output.

**Fig. 5:**
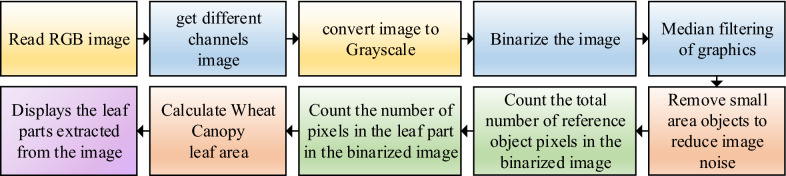
Process of calculating wheat canopy leaf area

When calculating the leaf area of wheat canopy, this paper selects the leaf area extraction method based on reference and the wheat leaf area is equal to the number of pixels × Unit pixel area [20]. Determine the unit pixel area in the RGB image with the help of the reference, and select the green paper consistent with the inner diameter of the round root box (d = 15 cm) as the test reference to simulate the green crop. As shown in Fig. 5, the process of processing RGB image based on MATLAB. Firstly, the RGB image of wheat canopy is read, and the image is processed based on R, G, B and color combination channels to obtain the gray image and gray histogram under different color channels [21]. The valley bottom gray value with obvious double peaks in the gray histogram is selected as the threshold for image binarization; In order to eliminate the influence of the environment on the image acquisition process, the median filter is used for image restoration and small target removal area; Then mark the processed image with a reference, count the total number of pixel points P_1_ of the reference in the binary image at this time, and then count the total number of pixel points P of the leaf part. Next, calculate the leaf area s of the wheat canopy according to formula (1).

1 $$S=\frac{{S}_{1}}{{P}_{1}}P$$

S in the above formula represents the total area of wheat leaves calculated; S_1_ is the reference area (S_1_ = 176.625cm^2^ in this paper after calculation); P is the total number of pixels contained in the blade image; P_1_ is the total number of pixels contained in the reference image.

#### Wheat phenotypic parameter extraction based on depth camera

Through the operator interface of the crop phenotype acquisition system, the depth camera is controlled to acquire wheat images. The raw image data includes depth image, infrared image (IR) and color image (RGB). Depth image = RGB image + depth map. The pixel value of the point in the depth image represents the distance (depth value) from the sensor to the object. Since there is a one-to-one correspondence between the RGB image and the pixel points in the depth map, we align the depth image with the RGB image by the correspondence of the pixel point coordinates.

**Fig. 6:**
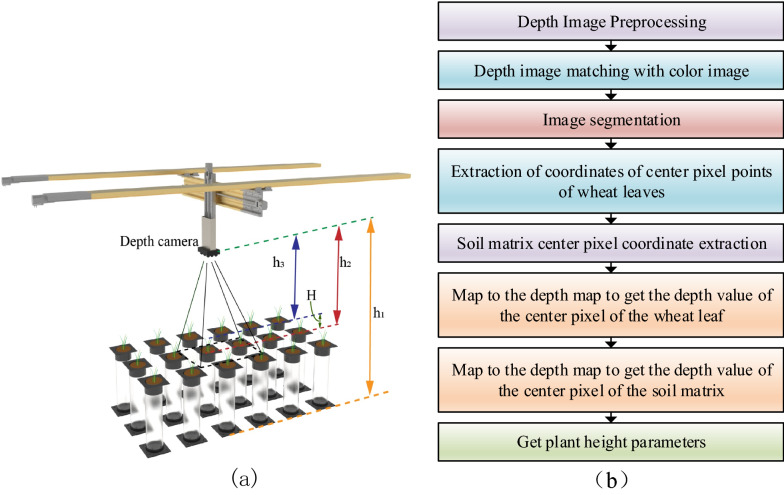
**a** Schematic diagram of crop plant height calculation; **b** Image processing flow of obtaining crop plant height

Plant height is defined as the distance between the aboveground part of the crop plant from the main stem root to the leaf. The principle of obtaining crop plant height based on depth image is shown in Fig. 6a, and the vertical distance extraction method from depth camera to crop leaves and soil matrix is shown in Fig. 6b. Combining the color image and depth image, extract the depth value of the central pixel of wheat leaf and the central pixel of soil matrix. The area where the wheat leaves meet (i.e., the main stem) was defined as the center of the leaves. The area of the soil matrix near the roots of wheat was considered as the center of the soil matrix. At the same time, measure the actual height of the round root box as a reference comparison, so as to determine the depth value of the depth camera from the ground is h_1_, the distance from the depth camera to the soil matrix surface is h_2_, and the distance from the depth camera to the crop leaf is h_3_. Then the calculation formula of plant height (H) is:

2 $$H = h_{2} - h_{3}$$

First, extract the center pixel coordinates (x, y) of the center of the wheat leaf and the soil matrix. Due to the interference of the environment or other factors, the raw data obtained by the depth camera will have noise, and it is necessary to perform denoising processing such as median filtering on the original depth image. Since the object of this paper is the plant height of a single wheat plant, image segmentation is required, and the image is segmented by region extraction, leaving only the target region for coordinate extraction of the target location (the center pixel point of the leaf of wheat and the center pixel point of the soil substrate). Second, match the RGB image to the depth image and extract the depth value of the center pixel. Since there is a one-to-one mapping relationship between the depth image and the color image, the position coordinates (x, y) of the leaf center pixel point and the soil matrix center point coordinates determined in the color image are imported into the depth image, and the corresponding coordinates in the depth image are imported into the depth image. The pixel position matrix M is matched, Z_1_ can be obtained from the leaf center pixel coordinate matrix M_1_, Z_2_ can be extracted from the soil matrix center pixel coordinate matrix M_2_, and the depth value Z_1_ corresponding to the leaf center pixel coordinate is the depth from the camera to the crop. The distance between the leaves is h_3_, and the depth value Z_2_ corresponding to the coordinates of the center pixel point of the soil matrix is ​​the distance between the depth camera and the crop leaves, which is h_2_. After extraction, make the difference for multiple times to get the average value; output the plant height parameter of the crop.

#### Extraction of wheat phenotypic parameters based on thermal imaging camera

**Fig. 7:**
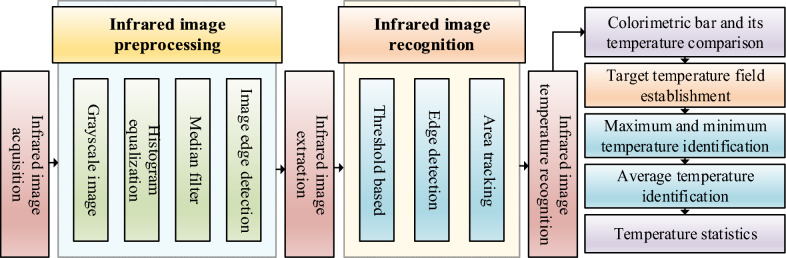
Infrared image processing flow

As shown in Fig. 7, firstly, the infrared image is gray transformed. Due to the one-to-one correspondence between gray value and temperature, 0 ~ 255 Gy values are used to represent the temperature distribution state in the image. According to the temperature value of pixel points, the temperature interpolation method is used for data processing and gray image is generated. The collected infrared thermal image is preprocessed by histogram equalization and median filtering. The purpose is to enhance the thermal image effect and remove the noise caused by environmental factors in the original infrared image. In order to obtain the canopy temperature of crops, it is necessary to identify the target crops in the infrared image, separate the wheat canopy area from the surrounding environment, and extract the wheat canopy area. Due to the irregular edges of wheat, there is interference from environmental factors such as soil matrix. We often use segmentation methods based on threshold and edge detection to divide the infrared image into several parts, separate the wheat canopy area from the surrounding environment, and extract the wheat canopy area. The purpose is to remove unnecessary information on the original image to minimize non-target interference. In the regional range of crop canopy, count the number of pixels in the wheat canopy area, extract the gray value corresponding to each pixel, count the canopy area temperature according to the corresponding relationship between gray value and temperature value, and establish the temperature distribution field of wheat canopy area [22]. Identify the highest and lowest temperature, divide the statistical temperature value by the number of pixels to obtain the average temperature of wheat canopy, and output the wheat canopy temperature parameters in the software interface.

#### Data collection

We selected four key periods in wheat growth to collect images for phenotypic study. The systematic measurements of wheat canopy phenotype at the trefoil stage, pre-tillering stage, late tillering stage and jointing stage were obtained automatically through the crop phenotype collection system. Manual measurements of wheat canopy leaf area size, plant height, and canopy temperature were obtained using graph paper [23, 24], a ruler, and a handheld infrared camera. The manual measurement time is consistent with the image data acquisition time of the crop phenotype monitoring system. The measurement tools used are: ① Vernier caliper (range 0-20 cm, measurement accuracy 1 mm); ②Ruler (range 0-50 cm, accuracy 1 mm); ③RAYTEK ST80 + industrial temperature measuring gun FLUKE handheld infrared thermometer, the detailed parameters are shown in Additional file 1: Table S6.

a. Leaf area measurement: Lay the leaf to be tested on the square paper (unit square area 1mm^2^), trace the outline along the edge of the leaf with a pencil, then count the number of squares occupied by the leaf shape, and the number of squares counted is for the leaf area, each wheat plant was measured three times and the average value was taken.

b. Plant height: In accordance with the guidance of agronomy breeding experts and relevant standards, the vertical length from the soil matrix to the highest point of the wheat leaf is measured manually with a ruler to be the plant height. Collect and record the plant height parameters of wheat in each growth period. Likewise, each wheat plant was measured three times for plant height and averaged.

c. Canopy temperature: Manually collect wheat canopy phenotype data with the help of a hand-held thermometer. Wheat canopy areas were collected using a Raytek ST80 + handheld thermometer. In order to make the measured canopy temperature data more representative, we measured three times at each canopy temperature collection point, and took the average of the three measurement results to represent the wheat canopy temperature value at the data collection point.

#### Data processing

We performed statistical analysis of the collected data using SPSS statistical software (IBM SPSS Statistics 26, Inc., Chicago, IL, USA). First, we analyzed the correlation between the two data sets, using the correlation coefficient(r) to evaluate the degree of correlation between the image eigenvalues and the wheat agronomic parameters (if it presents significance, the results are marked with an * in the upper right corner). In general, when r is greater than 0.7 it indicates a very strong relationship; r between 0.4 and 0.7 indicates a strong relationship; r between 0.2 and 0.4 indicates an average relationship [25].

In addition, we performed regression analysis of the systematic and manual measurements of wheat phenotypic data [26]. Regression models between systematic and manual measurements of leaf area, plant height and canopy temperature of wheat were developed. The goodness of fit and error between wheat phenotypic parameters based on systematic and manual measurements were assessed with the help of two metrics, coefficient of determination(R^2^) [27] and root mean square error (RMSE) [28].

## Results

### Correlation analysis

#### Correlation analysis of wheat leaf area

**Fig. 8:**
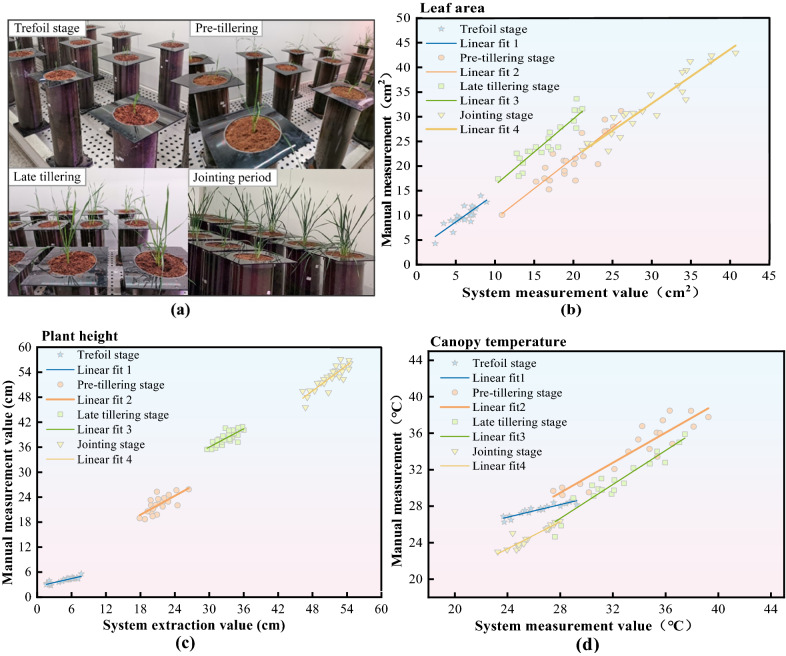
**a** Wheat growth stage; (**b-d**). Linear fitting between systematic and manual measurements of phenotypic characteristic parameters in wheat growth period

Table S7 shows the results of correlation analysis between systematic and manual measurements of leaf area of wheat. It can be seen that the systematic measurements of wheat leaf area were significantly and positively correlated (at the p < 0.05 level) with the manual measurements in the four growth periods of wheat, and the correlation coefficients were obtained to be greater than 0.84. On this basis, linear regressions were fitted to the two sets of data, and the evaluation indexes were R^2^ and RMSE. where R^2^ was used as the goodness-of-fit coefficient, reflecting the goodness-of-fit between the systematic measurements of wheat leaf area and the manual measurements. The larger the R^2^, the better the fit of the model, and the RMSE is used to measure the deviation between the systematic and manual measurements, which can better reflect the precision of the measurement. the smaller the RMSE (tends to 0), the better the regression. The fitting results are shown in Fig. 8 andAdditional file 1: Table S8, both for the linear regression model. The calculated R^2^ was greater than 0.71, and the goodness of fit between the system measurements and manual measurements of wheat leaf area performed well. the RMSE were less than 2.4 cm^2^. among them, the crop phenotype collection system showed the smallest RMSE for wheat leaf area measurements at the trefoil stage, which could be as small as 1.112 cm^2^. Therefore, the growth model of wheat leaf area could be monitored by the crop phenotype system for prediction.

#### Correlation analysis of wheat plant height

We correlated the systematic measurements of wheat plant height with the manual measurements and the results are shown in Additional file 1: Table S7. It can be seen that the correlation coefficients were all greater than 0.72. The systematic measurements of wheat plant height were significantly and positively correlated with the manual measurements (at the p < 0.05 level) in all four growth periods of wheat. On this basis, linear regressions were fitted to the two data sets, evaluating the indicators R^2^ and RMSE. the results of the fits are shown in Fig. 8 and Additional file 1: Table S8, and the equations are shown as linear regression models. The calculated R^2^ was greater than 0.83, indicating a good regression fit between the systematic and manual measurements of wheat plant height [29, 30]. The RMSEs in the fit results were all less than 2.5 cm. where the crop phenotype collection system showed the smallest RMSE in the wheat height measurements at the trefoil stage, the smallest could reach 0.349 cm, and the growth model of wheat plant height could be predicted by the crop phenotype monitoring system.

#### Correlation analysis of wheat canopy temperature

As shown in Fig. 8 and Additional file 1: Table S7. The correlation coefficients r between the systematic and manual measurements of wheat canopy temperature were greater than 0.92 during the wheat reproductive period, and both were significantly and positively correlated. The fitting results are shown in Fig. 8 and Additional file 1: Table S8. The R^2^ was greater than 0.84, and the RMSE was less than 1.3℃. The crop phenotype acquisition system exhibited the smallest measurement error of the wheat canopy temperature during the trefoil stage, and the smallest RMSE can reach 0.261℃. The results showed a significant correlation between the canopy temperature system-measured values obtained by the crop phenotype monitoring system and manual measurement values. A good fit was also observed. The crop phenotype monitoring system can be used to further study the water stress of wheat [31].

#### Dynamic changes of wheat phenotypic parameters under different nitrogen application rates

##### Dynamic changes of wheat leaf area and green index under different nitrogen application rates

**Fig. 9:**
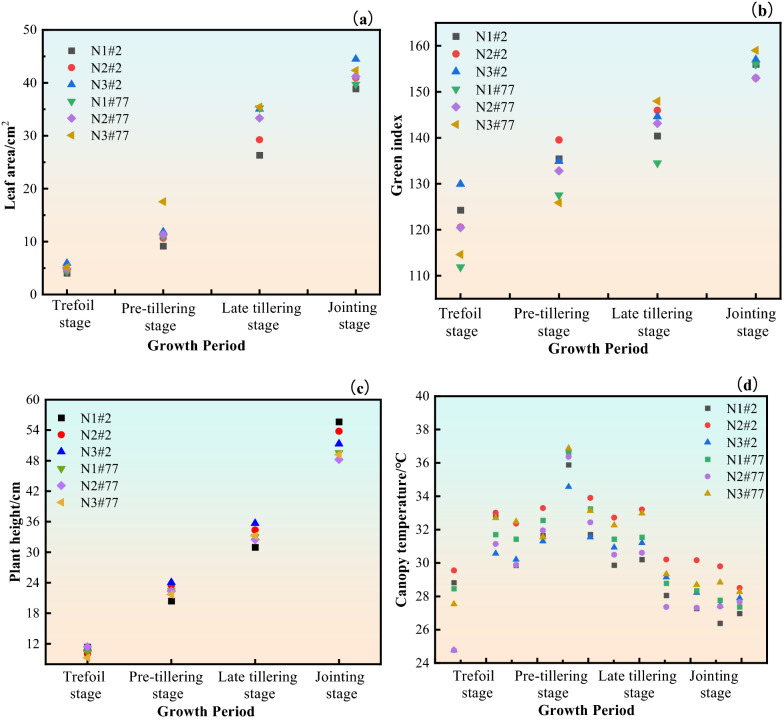
Dynamic changes of characteristic parameters of wheat during growth period under different nitrogen fertilizer concentration treatments

Fig. 9a, b show the dynamic changes of leaf area and green index of wheat during the growth period of wheat under different nitrogen fertilizer concentrations. In general, the effect of N fertilizer application on both wheat varieties tended to be the same. In the same growth period of wheat, different nitrogen fertilizer application rates have certain influence on the characteristic parameters of wheat canopy. In the same growth period, the leaf area and green index of wheat canopy increased with the increase of nitrogen fertilizer application, showing a certain sensitivity. The effect of different N fertilizer use on wheat leaf area was most obvious at the late tillering stage. for QN2 wheat, the average leaf area of wheat was 26.32 cm^2^ under N1 treatment, 29.25 cm^2^ under N2 treatment and 35.05 cm^2^ under N3 treatment. the increase in N fertilizer application promoted wheat canopy growth significantly.

The average leaf area of wheat increased from 11.9 cm_2_ to 41.3 cm^2^ from the pre-tillering stage to the jointing stage. Analysis of the reasons shows that the use of nitrogen fertilizer is conducive to the growth of wheat leaves, and the leaf color is dark green. Within the range, the more nitrogen fertilizer was applied to wheat, the number of tillers increased and the leaves grew vigorously [32]. With the advancement of the wheat growth period, the leaf area and green index of wheat under different nitrogen application rates showed an increasing trend.

#### Dynamic changes of wheat plant height under different nitrogen application rates

The dynamic changes of wheat plant height during the growth period are shown in Fig. 9c. For wheat at different growth stages under the same nitrogen application level, the plant height showed an increasing trend. The increase of wheat plant height was larger from the trefoil stage to the late tillering stage. The plant height increased from 10.58 cm to 33.28 cm, with an increase of 22.7 cm.While the increase of the plant height from the late tillering stage to the jointing stage was relatively slow, increasing only 16 cm. For wheat under different nitrogen fertilizer application conditions in the same growth period, the plant height showed an increasing trend with the increase of nitrogen fertilizer application concentration. For example, in the pre-tillering stage, the average plant height of wheat in the N3 treatment was 3.6 cm higher than that of wheat in the N1 treatment. But the increase trend of plant height was not obvious as the growth period went on. It can be seen that nitrogen fertilizer has a significant promoting effect on wheat plant height in the early stage of wheat growth. Within the application concentration range of 80 kg/hm^2^ ~ 240 kg/hm^2^, the higher the nitrogen fertilizer concentration, the more obvious the promotion of plant height growth.

#### Dynamic changes of wheat canopy temperature under different nitrogen application rates

The dynamic changes of wheat canopy temperature during the growth period are shown in Fig. 9d. The canopy temperature of the two different varieties of wheat in the same period was not very different. For the wheat under the same nitrogen fertilizer application level, the canopy temperature showed a trend of first increase and then decrease with the growth period, and the wheat canopy temperature increased from the trefoil stage to the late tillering stage. In the pre-tillering stage, the canopy temperature can be as high as 38.5℃.But from the late tillering stage, the temperature of wheat canopy gradually decreased. During the jointing stage, the temperature decreased to 24.39 ℃, which was due to the fact that nitrogen application could reduce the canopy temperature [33]. The variation of wheat canopy temperature under different N fertilizer application conditions during the same growth period was that the canopy temperature of wheat with higher N fertilizer application concentration was lower, and the canopy temperature of wheat under N1 treatment > N2 treatment > N3 treatment. The canopy temperature of wheat under N3 treatment was on average 1.3℃ lower than that under N1 treatment. This is in agreement with the findings of Yang, D. et al. [34].

## Discussion

With the development of phenomics nowadays, in order to achieve high-throughput, high-efficiency and high-precision phenotypic parameter acquisition, scholars of phenotypic research at home and abroad have focused their research on the development of phenotypic platforms, striving to develop new phenotypic platforms that meet the needs of phenotypic development [35]. We have summarized the advantages and disadvantages of different types of phenotyping platforms at present after research, as shown in Additional file 1: Table S9.

In order to make up for the limitations of climate and location when most phenotype platforms are used, as well as the shortcomings of traditional artificial climate box phenotype collection and analysis, continuous high-throughput collection of phenotypes during crop growth period is realized. The intelligent artificial climate chamber developed in this study has the functions of crop cultivation management and phenotype acquisition during the wheat growth period. We have developed an environmental control system that can set indoor temperature, humidity, light and other parameters, and designed a crop phenotype monitoring system composed of a high-precision mechanical transmission and multiple sensors. We also developed a phenotypic feature extraction and management software system.

We carried out a wheat cultivation experiment in an intelligent artificial climate chamber, selected different wheat varieties and applied different concentrations of nitrogen fertilizer, and collected image information of wheat samples with the help of the crop phenotype monitoring system. Correlation analysis was performed between systematic measurements of layer temperature and manual measurements. The results showed that the systematic measurement values of wheat canopy phenotype parameters based on the crop phenotype monitoring system were linearly correlated with the artificial measurement values, and the fitting degree was good. The effect of nitrogen fertilizer application on wheat canopy growth. Under the nitrogen fertilizer application of 80 kg/hm^2^ ~ 240 kg/hm^2^, with the increase of nitrogen fertilizer concentration, the wheat leaf area, plant height and canopy temperature decreased.

However, this study still has the following limitations. First of all, in the cultivation experiment in this paper, we focus on the difference in the response of wheat to nitrogen fertilizer concentration, and do not consider the influence of other factors on wheat growth, which is an ideal state. In actual production, factors such as pests and diseases, freezing damage do exist. In future research, the intelligent artificial climate chamber can further simulate the growth environment of wheat under different biotic or abiotic stresses, explore the growth differences of wheat and carry out phenotypic data research, so as to screen out excellent stress-resistant genes. We believe that it has great application potential.

Secondly, to explore the interaction mechanism between wheat phenotypes and the environment and genotypes, more phenotypic data information needs to be analyzed. Due to the long growth cycle of wheat and the impact of the new crown epidemic, we have not been able to carry out the full growth period of wheat. canopy image acquisition and phenotypic parameter acquisition, more experimental parameters need to be acquired in the future to continuously optimize the accuracy of crop phenotype analysis models. At the same time, the crop phenotype acquisition software developed in this paper preliminarily meets the requirements of image acquisition and phenotype analysis, and its functions can be further expanded and improved according to needs.

Therefore, our future work will include continuously enriching the functions of the crop phenotype acquisition system, deeply mining the data information contained in the depth images to extract more wheat phenotype parameters, and realizing the data information fusion of multiple sensors. In addition, experiments on various influencing factors of biotic and abiotic stress were carried out in the intelligent artificial climate chamber.

## Conclusion

In this study, we developed an intelligent artificial climate chamber for wheat cultivation and phenotyping. Compared with the current phenotypic platform, its use is not restricted by the climatic environment and place, and has the advantages of being movable, relatively low in construction cost and easy to promote. We have completed the overall design of the intelligent artificial climate chamber, the construction of the internal hardware system and the development of the software system. And with the help of wheat cultivation experiments, the feasibility verification of intelligent artificial climate chamber was completed. We found that continuous non-destructive measurements of wheat during the growing season can be achieved with the help of an intelligent artificial climate chamber. At the same time, cultivation experiments confirmed that increasing nitrogen fertilizer concentration can promote the growth of wheat stems and leaves and reduce leaf temperature.

Overall, the intelligent artificial climate chamber provides a high-throughput phenotyping research platform and a solution for crop breeders. The intelligent artificial climate chamber we developed makes up for the high cost of building large phenotyping platforms and the difficulty of scaling them up, and achieves the research goal of a low-cost and easy-to-scale-out facility that can be used regardless of climate and site constraints and has both crop cultivation and phenotype collection functions. It also improves the lack of phenotype data collection function of most traditional artificial climate chambers. Therefore, the intelligent artificial climate chamber is expected to be a powerful tool to assist crop breeders by in-depth study of the interaction mechanism between wheat phenotype and genotype and environment.

## Supplementary Information

**Additional file 1: Table S1** Test condition parameters of wheat cultivation in the intelligent artificial climate chamber **Table S2** Main environmental factor regulation parameter table **Table S3** Main equipment parameter table of environmental control system **Table S4** Main equipment parameters of high-precision mechanical transmission device **Table S5** Parameters of imaging camera in the crop-phenotype acquisition system **Table S6** Technical Parameters of Handheld Infrared Thermal Imager **Table S7** Correlation analysis of systematic and manual measurements of phenotypic characteristic parameters in wheat growth period **Table S8** Comparison of fitting analysis between the systematic and manual measurement values of phenotypic characteristic parameters in wheat growth period **Table S9** The advantages and disadvantages of this system are compared with other platforms

## Data Availability

Not applicable.
