# Supplementary material for: Evaluation of an intelligent artificial climate chamber for high-throughput crop phenotyping in wheat
Source: Plant Methods. 2022 Jun 7;18:77. doi: 10.1186/s13007-022-00916-9 (PMC9170875; doi:10.1186/s13007-022-00916-9)
Supplement: Supplementary file 1 — Additional file 1: Table S1 Test condition parameters of wheat cultivation in the intelligent artificial climate chamber Table S2 Main environmental factor regulation parameter table Table S3 Main equipment parameter table of environmental control system Table S4 Main equipment parameters of high-precision mechanical transmission device Table S5 Parameters of imaging camera in the crop-phenotype acquisition system Table S6 Technical Parameters of Handheld Infrared Thermal Imager Table S7 Correlation analysis of systematic and manual measurements of phenotypic characteristic parameters in wheat growth period Table S8 Comparison of fitting analysis between the systematic and manual measurement values of phenotypic characteristic parameters in wheat growth period Table S9 The advantages and disadvantages of this system are compared with other platforms [file 13007_2022_916_MOESM1_ESM.docx]

**Supplementary Information**

**Table S1 Test condition parameters of wheat cultivation in the intelligent artificial climate chamber**

| **Fertilization**  **/Water volume** | **Nitrogen fertiliser（N1）** | **Nitrogen fertiliser（N2）** | | **Nitrogen fertiliser（N3）** | **Potash fertiliser** | **Phosphate**  **Fertiliser** | | **Irrigation volume** | |
| --- | --- | --- | --- | --- | --- | --- | --- | --- | --- |
|  | 80 kg/hm^2^ | 160 kg/hm^2^ | | 240 kg/hm^2^ | 120 kg/hm^2^ | 120 kg/hm^2^ | | 450 m^3^/hm^2^ | |
| Environmental conditions | **Period** | **CO_2_** | | **Humidity** | **Illumination** | **Air pressure** | **Temperature** | | |
|  | Growth Period | 500 PPM | | 70% RH | Day100%  Night30% | 500BAR | Day (25 ℃) | | Night (18 ℃) |
| Acquisition time | Trefoil stage | | 27 November 2020  28 November 2020  29 November 2020 | | | 9:00–9:30  13:00–13:30  16:00–16:30 | | | |
|  | Pre-tillering stage | | 17 December 2020  18 December 2020  19 December 2020 | | | 9:00–9:30  13:00–13:30  16:00–16:30 | | | |
|  | Late tillering stage | | 19 January 2021  20 January 2021  21 January 2021 | | | 9:00–9:30  13:00–13:30  16:00–16:30 | | | |
|  | Jointing stage | | 17 March 2021  18 March 2021  19 March 2021 | | | 9:00–9:30  13:00–13:30  16:00–16:30 | | | |

**Table S2 Main environmental factor regulation parameter table**

| **Environmental parameters** | **Adjustment range** |
| --- | --- |
| Temperature | 0 ℃–50 ℃ |
| Illumination | 0–3000 Lux |
| Air pressure | 0.03-0.1MPa |
| Humidity | 10%–80% RH |
| CO_2_ concentration | 1000–1500 PPM |

**Table S3 Main equipment parameter table of environmental control system**

| **Device name** | **Model** | **Core Parameters** | **Features** |
| --- | --- | --- | --- |
| HMI | Delta HMC08-N500S52 | 800 x 480 (pixels) | Stable operation and friendly operation |
| PLC | Delta DVP-EH3 PLC | 4 axes 200 kHz, 32-bit CPU + ASIC | Good stability and powerful computing power |
| Air pressure sensor | DLK301 | ±0.5%FS、±1%FS | High-sensitivity imported probe, accurate data monitoring |
| Temperature Sensor | WZP-PT100-230 | ±(0.15%+0.002%) | Platinum thermal resistance, wide temperature range and high precision |
| Humidity Sensor | LR9502 | ±3%RH（5%RH~95%RH，25℃） | Small volume specification, high measurement accuracy, easy operation |
| CO_2_ sensor | BMG-CO_2_-NDIR | ±5%； | Protection type, not affected by external high humidity environment |
| Copeland Hermetic Compressor | ZB38KQE | 5.0HP,50Hz | High volumetric efficiency and high adiabatic efficiency |
| Digital display light touch temperature controller | XMTG-3002 CU50 | ≤0.75%FS | Operational amplifier signal acquisition, high control accuracy |

**Table S4 Main equipment parameters of high-precision mechanical transmission device**

| **Equipment** | **Model** | **Core Parameters** |
| --- | --- | --- |
| HMI | DOP-107EV | 400cd/m^2^，COM1:RS-232, COM2:RS-232/485, COM3:RS-422/485 |
| PLC | DVP-SV2 | 4 axes 200 kHz,32-bit CPU + ASIC |
| server Driver | ASD-B2-0421-B | 400W/220V，Single/three-phase,550HZ |
| servo motor | ECMA-C20604RS | pixels: 17 bit (160000 ppr) |
| Precision rolling guide pair | GGB25IAA | ±20μm (Accuracy) |

**Table S5 Parameters of imaging camera in the crop-phenotype acquisition system**

| **Sensor type** | | 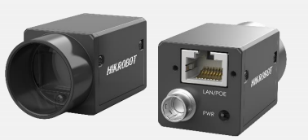  **RGB camera** | 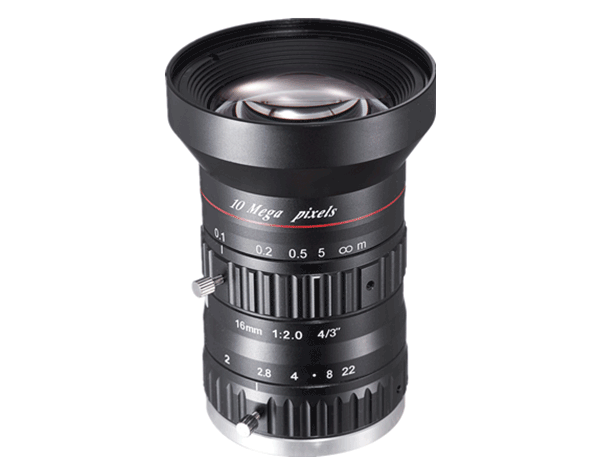  **RGB camera lens** | 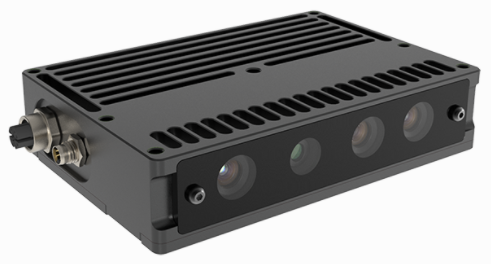  **Depth camera** | 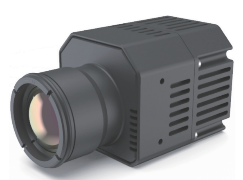  **Thermal imaging cameras** |
| --- | --- | --- | --- | --- | --- |
| Type | LT384H/640H | SA1620 M-10MP | FM850-GI-E1 | LT640H |  |
| Detector Type | CMOS | - | RGB-D+IR | VOX uncooled infrared focal plane |  |
| Resolution | 2592×2048 | - | 1280 x 960 @ 15 fps  2592 x 1944 @ 6 fps | 640×512 |  |
| Interface | Gigabit Ethernet | C-mount | USB2.0 | RS-232/UART（3.3V） |  |
| Operating temperature | 0~50℃ | -10~50℃ | 0~45℃ | 0℃~60℃ |  |
| Typical power consumption | <3.3W | - | <5.2W | <2W |  |
| Functional Features | Excellent image quality, fast real-time transmission, and support for multiple exposure modes | High resolution, excellent imaging quality, high transmittance, good stability | Active binocular, strong anti-interference ability, support trigger mode | High temperature measurement accuracy, support for secondary development, high frame rate |  |

**Table S6 Technical Parameters of Handheld Infrared Thermal Imager**

| **Model** | **Display resolution** | | **D：S** **(distance: measuring spot diameter)** | **IR spectral response** | **Infrared response time** | **Temperature measurement range** | **Measurement accuracy** |
| --- | --- | --- | --- | --- | --- | --- | --- |
| Raytek ST80+ | | 0.1℃  /0.1℉ | 50：1 | 8~14μm | ＜500ms | -32~750℃ | ±0.2℃ |

**Table S7** Correlation analysis of systematic and manual measurements of phenotypic characteristic parameters in wheat growth period

| **Characteristic Parameters** | **Growth Period** | **N** | **P value** | **Pearson’ r** |
| --- | --- | --- | --- | --- |
| Leaf area  （cm^2^） | Trefoil stage | 23 | 3.57236E-7 | 0.84645* |
|  | Pre-tillering stage | 22 | 1.03253E-9 | 0.92242* |
|  | Late tillering stage | 23 | 1.29195E-8 | 0.89019* |
|  | Jointing stage | 23 | 4.5663E-12 | 0.94979* |
| Plant height  （cm） | Trefoil stage | 26 | 1.00043E-10 | 0. 84821* |
|  | Pre-tillering stage | 23 | 1.41311E-4 | 0.72357* |
|  | Late tillering stage | 26 | 2.50211E-6 | 0.78098* |
|  | Jointing stage | 26 | 5.0194E-9 | 0.87496* |
| Canopy temperature  （℃） | Trefoil stage | 18 | 2.86352E-8 | 0.92809* |
|  | Pre-tillering stage | 19 | 2.28567E-8 | 0.92084* |
|  | Late tillering stage | 18 | 2.20982E-9 | 0.94821* |
|  | Jointing stage | 18 | 2.06348E-8 | 0.93106* |

(Note: *at the 0.05 level (two-tailed), correlation is significant.)

**Table S8** Comparison of fitting analysis between the systematic and manual measurement values of phenotypic characteristic parameters in wheat growth period

| **Characteristic Parameters** | **Growth Period** | **Fitting equation** | **R^2^** | **RSME** |
| --- | --- | --- | --- | --- |
| Leaf area  （cm^2^） | Trefoil stage | y=1.125x+3.043 | 0.7165 | 1.112 |
|  | Pre-tillering stage | y=1.344x+2.681 | 0.8509 | 1.811 |
|  | Late tillering stage | y=1.238x-3.146 | 0.7924 | 2.365 |
|  | Jointing stage | y=1.098x-0.238 | 0.9021 | 2.046 |
| Plant height  （cm） | Trefoil stage | y=0.5002x+3.833 | 0.8397 | 0.349 |
|  | Pre-tillering stage | y=0.823x+2.443 | 0.8464 | 1.971 |
|  | Late tillering stage | y=1.228x-1.799 | 0.8452 | 2.139 |
|  | Jointing stage | y=0.8273x+2.013 | 0.8492 | 2.429 |
| Canopy temperature（℃） | Trefoil stage | y=0.571x+13.93 | 0.8613 | 0.261 |
|  | Pre-tillering stage | y=0.8234x+6.416 | 0.8479 | 1.285 |
|  | Late tillering stage | y=0.9306x+0.604 | 0.8991 | 0.930 |
|  | Jointing stage | y=0.7405x+5.564 | 0.8669 | 0.436 |

**Table S9.** The advantages and disadvantages of this system are compared with other platforms

|  | **Platform Type** | **Advantages** | **Disadvantages** |
| --- | --- | --- | --- |
| **Indoor** | Conveyor type | High transmission efficiency, unaffected by harsh outdoor environments | High upfront investment and maintenance costs make it difficult to scale up; it can only be used on a fixed basis and cannot replace field phenotyping studies. |
|  | Orbital type | The operation process is relatively simple, and the site required for platform erection is small |  |
| **Outdoor** | Fixed Rail Type | Wide monitoring range, suitable for large scale phenotype data detection | High investment and construction costs and the need for regular maintenance; susceptible to disruptions such as weather and environmental factors. |
|  | Drone Type | High degree of freedom, wide monitoring range and large scale. | High requirements for imaging sensors; limited by the UAV's carrying weight and endurance. |
|  | Vehicle-mounted | Automatic navigation is available, which is less costly, reduces manpower and improves work efficiency. | Impact on imaging quality due to uneven road surface and equipment shake; affects crop growth. |
